# Analysis of heavy metal content in protein powders available on the Hungarian market: a reassuring snapshot, but not a reassuring quality guarantee

**DOI:** 10.1017/jns.2025.10024

**Published:** 2025-07-16

**Authors:** István László Horváth, Gyula Kajner, Gábor Galbács, Dezső Csupor

**Affiliations:** 1 Department of Pharmay Administration, University Pharmacy, Semmelweis University, Hőgyes Endre utca 7–9, 1092 Budapest, Hungary; 2 Center for Translational Medicine, Semmelweis University, Üllői út 26, 1085 Budapest, Hungary; 3 Department of Molecular and Analytical Chemistry, University of Szeged, Dóm Square 7–8, 6720 Szeged, Hungary; 4 Institute of Clinical Pharmacy, Faculty of Pharmacy, University of Szeged, 6725 Szeged, Hungary; 5 Institute for Translational Medicine, Medical School, University of Pécs, 7624 Pécs, Hungary

**Keywords:** Heavy metal: Inductively coupled plasma mass spectrometry, Laser-induced breakdown spectroscopy: Protein powder, DEA, dried egg albumin, EU, European Union, ICP-MS, inductively coupled plasma mass spectrometry, LIBS, laser-induced breakdown spectroscopy, MC, micellar casein, ND, not detectable, below the detection limit, NG, no gas mode, WPC, whey protein concentrate, WPH, whey protein hydrolysate, WPI, whey protein isolate, UK, United Kingdom, USA, United States of America

## Abstract

Amateur and professional athletes often consume protein supplements to accelerate muscle gain; however, it has been suggested that these products not only are associated with risks when consumed excessively. Several recent reports have indicated that certain products are contaminated with heavy metals. Therefore, in this study, we aimed to investigate protein powders in Hungary for heavy metal contamination. A total of 22 commercially available protein powders (including whey, vegan, and beef based) were purchased on the internet for testing. We analysed the samples using laser-induced breakdown spectroscopy (LIBS) and inductively coupled plasma mass spectrometry (ICP-MS) to assess heavy metal contamination. The products were analysed for the presence of 16 elements (Be, Al, Cr, Mn, Co, Ni, Cu, As, Se, Rb, Cd, Sb, Cs, Ba, Hg, and Pb). The LIBS spectral analysis revealed the characteristics of the protein elements (C, C_2_, H, N, and O) and alkaline metals (Ca, Na, K, and Mg), which were consistent with the previous results. Neither LIBS nor ICP-MS measurements detected significant heavy metal content in the investigated samples above the limit specified in the regulations. Heavy metal contamination of protein supplements can be a serious health threat. Based on the varied results of the previous studies, it is prudent to include testing for heavy metals as part of the routine and mandatory quality control of these products.

## Graphical abstract

**Figure:**
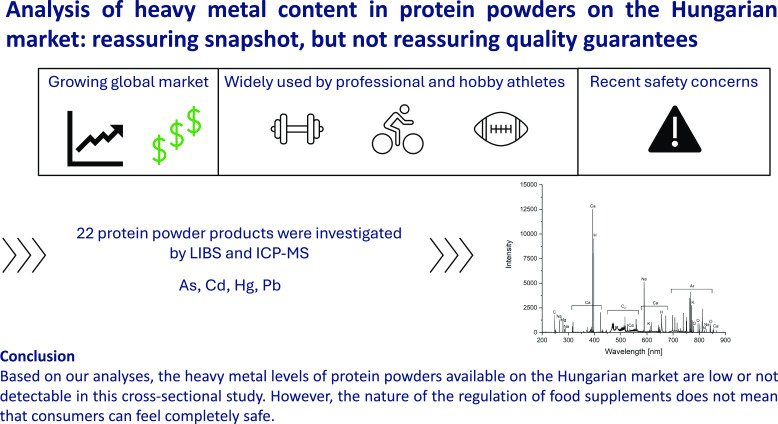

## Introduction

There is an increasing trend of mindful health in society, which includes healthy food consumption, special diets, and regular recreational activities. For the latter, many athletes use protein powders to improve lean and fat mass, muscular strength, and size^(1,2)^. The global market for protein supplements was valued at 5.83 billion USD in 2022 and has demonstrated continuous growth over the years. Most consumers of these products are located in North America and account for 41.4% of the total market^(3)^.

With numerous protein powder supplements having various formulations available on the market, based on the origin of the protein, they can be classified into animal- and plant-based products. The most convenient way to produce protein supplements is to use the by-products of cheese production, such as whey, which is the leftover liquid from the coagulation process. It can be formulated into whey protein concentrate, whey protein isolate, and whey protein hydrolysate. Casein, found in the solid part (curd) during production, can also be used to supplement protein intake^(4)^.

Animal-sourced proteins, sold as single-component products or mixtures with added WP, include egg whites (egg protein) and beef, which is processed in multiple steps. Plant-based protein products are derived from seeds with high protein content (most commonly soybean, chickpea, or rice) through various processes that are unique to each plant^(5)^.

Protein powders are ‘food intended for sportspeople’ according to the European Union (EU) regulation No. 609/2013 and are part of the ‘sport nutrition sector’ based on the COM/2016/0402 report. In general, the production of these supplements is governed by the horizontal rules of EU food law. Marketing and labelling of these products must include the branding, a clear description of the product, instructions, ingredients, and health and nutritional claims as permitted by Regulation 1924/2006^(6)^. Although food manufacturing in the EU is well-regulated, illegally imported products have no quality standards for production.

Although proteins are essential macronutrients, their excessive consumption might pose health risks. A high dietary protein intake can cause intraglomerular hypertension, resulting in kidney hyperfiltration and glomerular injury, while long-term high protein intake may result in the development of chronic kidney disease^(7)^. The recommended daily protein intake is 0.83 g/kg body mass per day^(8)^. Protein consumption exceeding 1.5 g/kg per day is generally considered to be a high-protein diet, which occurs in an unbalanced diet.

Targeted protein intake can be dangerous even when it does not exceed the recommended amount. Several studies conducted in the USA have shown detectable amounts of heavy metal contamination in whey protein supplements. The Clean Lab Project’s results indicated that of the 130 investigated supplements, 40% of the products contained elevated lead, mercury, cadmium, and arsenic levels. Around 75% of the tested products had detectable lead levels, which exceeded the safe level in 33% of the cases. Organic products had twice the level of heavy metal contamination^(9)^. Consumer Reports’ investigation of 15 products revealed that all of the tested supplements contained at least one of the studied elements (arsenic, cadmium, lead, or mercury) above the concentration level specified in the safety standards^(10)^. In addition, concerns were previously raised about the product labelling and the actual protein content of the products. Moreover, if the production factory is not maintained properly, the product could be contaminated with doping agents^(11)^. These results are from the USA. Although the United States Pharmacopeia as well as the EU regulates the limits of heavy metal impurities, compliance with these standards is voluntary for dietary supplement production^(12,13)^.

Prolonged exposure to concentrations of heavy metals results in adverse reactions and bioaccumulation^(14)^. The absorption of heavy metals through the gastrointestinal tract causes harmful effects, such as disrupted enzymatic activity, attachment to binding sites, or substitution for essential metal ions, which can lead to life-threatening conditions^(15)^. Higher mercury intake causes neurological and renal disorders, while cadmium and lead substitutes calcium in the bone, thus causing osteomalacia. Arsenic is toxic to the nervous system, leading to neurodegenerative diseases^(14,15)^. The health risk assessment by Bandara et al. claimed that despite the heavy metal content, protein powders pose no threat to public health if consumed at their recommended amounts^(16)^.

Heavy metal contamination can be attributed to manufacturing processes (by using deteriorated processing equipment or combining trace chemicals and solvents), animal feed, or the farms where the plants are grown. Cultivating plants in contaminated fields can lead to the accumulation of toxic elements^(17)^, thus contaminating herbal, especially, vegan products. Since naturally occurring plant proteins are less effective at stimulating muscle growth, consumer demand for protein supplements has been increasing^(18)^.

The above-mentioned elevated levels of heavy metal contents were reported by the studies conducted in the USA; however, to our knowledge, research is limited regarding heavy metal contamination of protein powder products in Hungary. The primary aim of this study was to analyse the metal (arsenic, cadmium, mercury, and lead) content of commercially available protein powders in Hungary using inductively coupled plasma mass spectrometry (ICP-MS) and laser-induced breakdown spectroscopy (LIBS). ICP-MS, a modern laboratory-based ultratrace analytical method with sub-ppb detection limits, is frequently used for the determination of toxic and essential elements in liquid food^(19,20)^. To enable liquid sample introduction, the samples must undergo an acid digestion procedure before analysis. On the other hand, LIBS is an emerging rapid trace analytical spectroscopy method used in the field of food and food additives inspection. Used directly on solid samples, it is a rapid technique with ppm-level detection limits. In addition, not only does LIBS analysis consume micrograms of samples but the instrument also so compact and robust that it can even be used in the field^(21,22)^.

## Methods

We purchased 22 commercially available protein powders based on popularity and content from online retail sellers to represent the overall market. The properties of the products are listed in Table 1. Samples were taken to analyse the metal content of the products.

**Table 1.** Main properties of the protein powders based on manufacturer’s specifications

Sample	Protein source	Additives	Sweetener	Flavour	Production
1	MC, glutamine	Soy lecithin	Sucralose, neohesperidin	Vanilla, safflower	Czech Republic
2	WPC, WPI, WPH, MC, DEA	Guar gum, soy lecithin, lactase, bromelain, papain	Steviol-glycosides	Oat	Czech Republic
3	WPC	Roughage inulin, silicon dioxide, bromelain, papain	Sucralose, neohesperidin	Vanilla, safflower	Czech Republic
4	WPC, WPI, WPH, MC, DEA	Soy lecithin, lactase, bromelain, papain	Sucralose, neohesperidin	Vanilla, safflower	Czech Republic
5	WPC, WPI, glutamine	Taurine, bromelain, papain, soy lecithin, xanthan gum, silicon dioxide	Acesulfame potassium, sucralose	Vanilla	EU
6	Pea, rice, coconut, pomegranate, pumpkin	Xanthan gum, vitamin B12, sodium chloride, silicon dioxide	Acesulfame potassium, sucralose	Vanilla	EU
7	WPC, WPI, WPH	Magnesium citrate, zinc oxide, vitamin E, Vitamin B6, bromelain	Sucralose, steviol-glycosides	Unflavoured	Germany
8	WPC	Sunflower lecithin	Steviol-glycosides	Vanilla	Germany
9	WPC, WPI, glutamine, arginine, leucin, isoleucine, valine	Soy lecithin, cellulose gum, sodium chloride, bromelain	Sucralose	Vanilla	USA
10	Pea, rice, glutamine, arginine	Sodium glycine, soy lecithin, quinoa flour, carrageen, cellulose gum, goji berry, acai berry, sodium chloride, cinnamon	Sucralose	Vanilla	USA
11	Pea, rice	Maltodextrin, sodium carboxymethyl cellulose, sodium chloride,	Acesulfame potassium, sucralose	Vanilla	EU
12	WPC, skim milk powder, whey powder, glutamine, leucine, isoleucine, valine	Bromelain, papain, lactase, inulin, silicium dioxide	Acesulfame potassium, sucralose	Vanilla	EU
13	Pea, rice	Guar gum, xanthan gum, potassium phosphate, sodium chloride	Sucralose	Vanilla	Belgium
14	WPC, WPI	Inulin, guar gum, xanthan gum, potassium phosphate, sodium chloride, coconut	Sucralose	Chocolate	Belgium
15	WPC	Soy lecithin	Sucralose	Vanilla	UK
16	Beef protein hydrolysate leucine, isoleucine, valine	Sodium carboxymethyl cellulose, potassium citrate, magnesium oxide	Steviol-glycosides, sucralose	Chocolate	Slovakia
17	Brown rice	Guar gum, sodium chloride	Steviol-glycosides	Vanilla	USA
18	Pea, pumpkin seed, brown rice, quinoa, lemna	Fitase, protease I and II, pineapple, papaya, cellulase, amylase, sodium chloride, coconut oil, probiotics, luo han guo	Steviol-glycosides	Chocolate	Canada
19	WPC	Sodium carboxymethyl cellulose	Sucralose	Vanilla	USA
20	WPC, WPI, WPH	Soy lecithin, xanthan gum	Sucralose	Vanilla	Germany
21	WPC, WPI	Sunflower lecithin, calcium-phosphate, xanthan gum, acacia gum, sodium chloride, silicium dioxide, amylase, protease, lactase, lipase, cellulase	Steviol-glycosides, sucralose	Vanilla	Czech Republic
22	WPC, glutamine, leucin, arginine, isoleucine, valine	Cocoa butter, coconut oil, milk powder, emulsifier, potassium phosphate, cellulose gum, sodium chloride, soy lecithin, glucose syrup, saccharose	Sucralose	Vanilla	EU

MC: micellar casein, WPC: whey protein concentrate, WPI: whey protein isolate, WPH: whey protein hydrolysate, DEA: dried egg albumin, EU: European Union, USA: United States of America, UK: United Kingdom.

### LIBS analysis

For the LIBS experiments, a sample of each protein powder was pressed into cylindrical (diameter = 13 mm, height = 4 mm) pastilles with an applied pressure of 1 tonne using a Specac® hydraulic press (Specac Inc. Orpington, UK). All the measurements were performed using a J-200 LA-LIBS tandem spectrometer (Applied Spectra Inc., USA) in LIBS mode, along with Axiom 2.0 instrument control software (Applied Spectra Inc., USA). The following settings were used for the measurements: 266 nm laser wavelength, 9.3 mJ laser pulse energy, 6 ns pulse duration, 200 µm spot diameter, 1.05 ms integration time, 0.5 µs gate delay, and an argon atmosphere. Ultraviolet–visible optical emission spectra were recorded between 190 and 1,040 nm with an optical resolution of 0.07 nm. For each pastille, 15 single-shot LIBS spectra were recorded from 15 individual, nonoverlapping measurement points. The average of the 15 spectra served as the basis for the elemental analysis of each protein powder. The spectral lines were identified using the built-in National Institute of Standards and Technology database of Clarity NeXt software (Applied Spectra Inc., USA).

### ICP-MS analysis

All the measurements were conducted using an Agilent 7700X (Agilent Technologies, USA) ICP-MS, operated with 1,550 W R.F. power, 10 mm sampling depth, and a flow rate of 15 L/min high-purity (99.996%) argon gas (Messer Hungarogáz Kft., Hungary). During the analysis, the sample uptake rate was set to 750 µL/min, and the data acquisition software was used in the analogue mode with an integration time of 1 s. Calibration standards were prepared using a 10 mg/L multi-element standard (Inorganic Ventures, USA) and 1,000 mg/L CertiPUR mono-element Hg and Sb standard solutions (Merck GmbH, Germany). The instrument was calibrated for all elements by using seven concentrations (0, 0.1, 0.5, 1, 5, 25, and 90 μg/L). The elements used as internal standards (Bi, In, Sc, and Y) during measurements were added to all the samples (both the calibration and protein samples) at 10 μg/L concentration using a 10 mg/L internal standard solution mixture (Inorganic Ventures, USA). High-purity (99.999%) He gas (Messer Hungarogáz Kft., Hungary) was used as collision gas to reduce the extent of polyatomic interferences for some isotopes (Table 2).

**Table 2.** The list of isotopes detected, and the settings used during their measurements

Element	Be	Al	Cr	Mn	Co	Ni	Cu	As	Se	Rb	Cd	Sb	Cs	Ba	Hg	Pb
Mass number	9	27	52	55	59	60	63	75	82	85	114	121	133	137	202	208
Collision cell mode	NG	NG	He	He	NG	NG	NG	He	NG	NG	NG	NG	NG	He	NG	He
Internal standard	Li	Sc	Sc	Sc	Sc	Sc	Sc	Y	Y	Y	In	In	In	In	Bi	Bi

NG = no gas mode, He = He gas mode.

The protein powders were thoroughly homogenised before the digestion process. Subsequently, three 100 mg aliquots were weighed (for the three repetitions) using a RADWAG, AS 82/220.X2-type analytical balance (RADWAG, Poland). Next, 3 mL of trace analytical-grade concentrated (67%) nitric acid (VWR Chemicals, USA) was added to each aliquot. After 60-min of digestion at 180°C, 1.5 mL of trace analytical-grade 30% hydrogen peroxide (VWR Chemicals, USA) was added at 180°C for 30 min. The cooled solutions were quantitatively filtered into a 50 mL volumetric flask through a hydrophilic polytetrafluoroethylene syringe filter with a nominal pore size of 0.2 µm using a water jet pump. After the addition of the internal standard, each sample was complemented with trace-quality de-ionised water obtained from a MilliPore Elix 10 device that was equipped with a Synergy polishing unit (Merck GmbH, Germany).

For the ICP-MS measurements, the signals of the elements selected for analysis were recorded using the following isotopes: ^9^Be, ^27^Al, ^52^Cr, ^55^Mn, ^59^Co, ^60^Ni, ^63^Cu, ^75^As, ^82^Se, ^85^Rb, ^114^Cd, ^121^Sb, ^133^Cs, ^137^Ba, ^202^Hg, and ^208^Pb. The signals of the internal standard elements were monitored using the ^6^Li, ^45^Sc, ^89^Y, ^115^In, and ^209^Bi isotopes. The signals of all these isotopes in all the samples were recorded by ICP-MS both with and without (No gas mode) using Agilent’s ORS (Octopole Reaction System) in collision cell mode (He gas) to decrease the abundance of polyatomic species, which cause spectral interferences (He gas mode). During the evaluation, a suitable measurement mode was selected for all the elements separately based on both theoretical considerations (plasma conditions and sample matrix composition) and experimental data, which are detailed in the following sections.

## Results

### LIBS analysis results

**Fig. 1.**
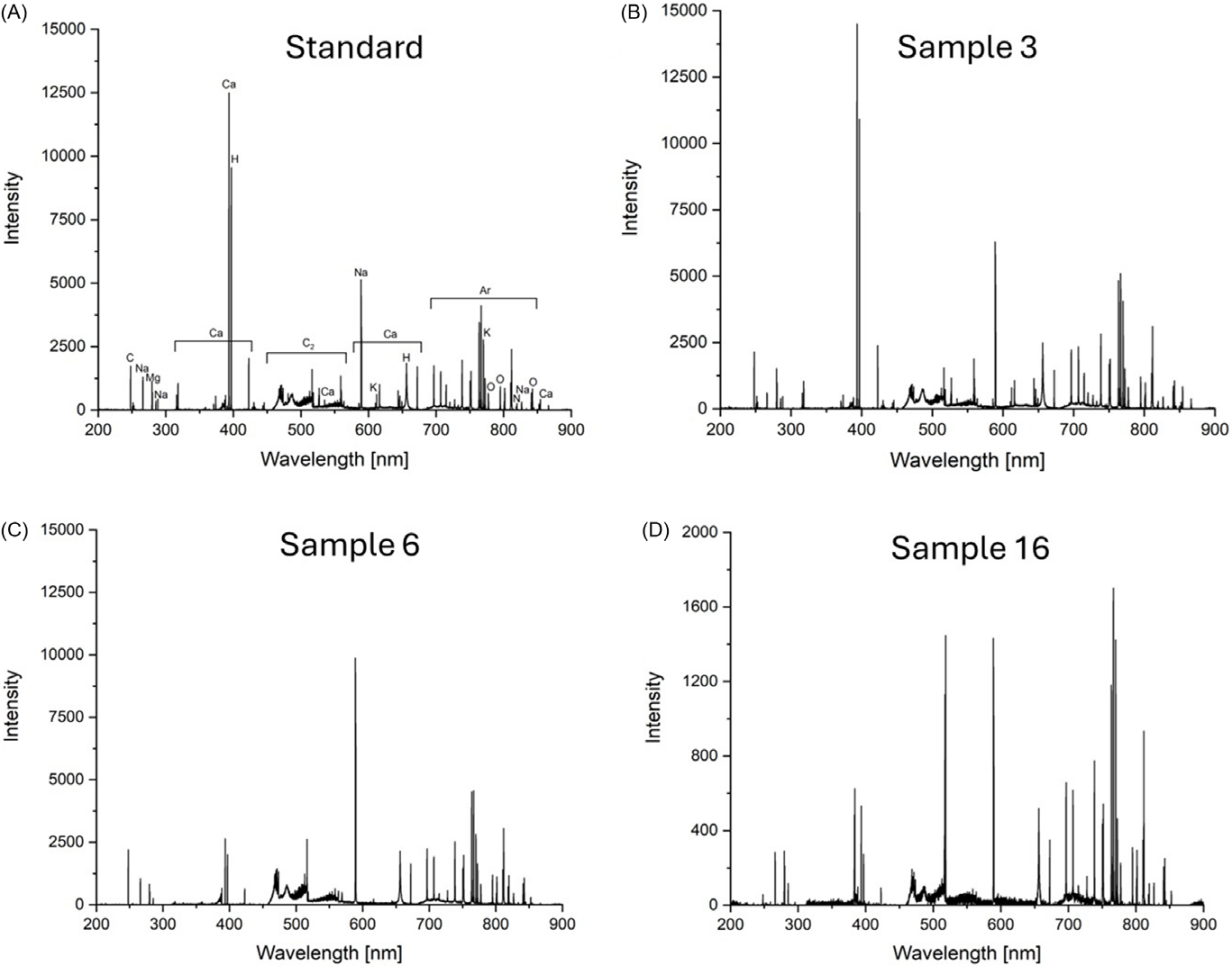
Laser-induced breakdown spectroscopy (LIBS) spectrum of A) sample 7 (whey-based), B) sample 1 (casein-based), C) sample 6 (vegan-based), and D) sample 16 (beef-based) protein powder.

LIBS spectral analysis revealed the characteristics of protein elements (C, C_2_, H, N, and O) and alkaline metals (Ca, Na, K, and Mg) (Fig. 1). The LIBS spectrum of each investigated sample is provided in the Supplementary material. An argon atmosphere, which was necessary for the analysis, resulted in Ar peaks, and no toxic elements were detected.

### ICP-MS analysis results

Several (mostly potentially toxic) elements were selected to assess the trace-elemental composition of the protein powders. A summary of our findings is listed in Table 3, excluding the elements Be, Cr, Co, Ni, As, Cd, Sb, Cs, Hg, and Pb, which remained under the detection limit and could not be quantified for all the powders examined.

**Table 3.** ICP-MS-determined metal concentration of the dry protein samples (powder-based ppm, mg/kg)

Sample	Al	Mn	Cu	Se	Rb	Ba
Whey proteins						
1	ND	ND	1,677 (1,254)	ND	1,810 (0,902)	1,375 (0,8889)
2	ND	4,396 (0,6429)	2,261 (1,117)	ND	4,568 (1,747)	ND
3	ND	ND	1,377 (0,757)	ND	4,029 (1,345)	ND
4	ND	ND	ND	ND	4,198 (1,302)	ND
5	ND	ND	ND	ND	4,718 (1,289)	ND
7	ND	ND	ND	ND	4,782 (1,907)	1,605 (0,7843)
8	ND	ND	2,582 (1,772)	ND	4,338 (1,407)	1,211 (0,8204)
9	ND	ND	3,167 (1,909)	ND	4,209 (1,971)	ND
12	6,419 (2,493)	4,160 (1,540)	6,321 (2,168)	ND	4,728 (2,241)	1,592 (1,059)
14	3,598 (0,9443)	4,070 (1,464)	4,325 (1,708)	ND	8,216 (0,744)	3,395 (1,337)
15	ND	ND	3,975 (2,185)	ND	4,358 (1,213)	ND
19	ND	ND	ND	ND	4,734 (1,030)	ND
20	ND	ND	ND	ND	4,351 (2,137)	ND
21	ND	ND	ND	ND	4,699 (2,523)	3,858 (2,501)
22	ND	ND	3,130 (2,246)	ND	4,723 (1,324)	ND
Vegan proteins						
6	4,955 (1,731)	8,152 (0,818)	4,780 (1,861)	ND	ND	ND
10	40,14 (2,323)	8,370 (1,230)	10,56 (1,129)	ND	1,175 (1,018)	2,099 (1,440)
11	11,01 (2,452)	15,433 (0,996)	10,22 (1,330)	ND	0,849 (0,616)	1,822 (0,5968)
13	11,07 (0,7170)	5,413 (0,598)	20,71 (1,380)	1,136 (0,159)	ND	3,504 (0,8150)
17	10,34 (1,552)	17,174 (0,503)	12,53 (2,211)	ND	ND	ND
18	23,04 (1,866)	20,875 (1,231)	11,71 (1,708)	ND	3,118 (1,236)	4,159 (0,9904)
Beef protein						
16	6,078 (1,963)	3,179 (1,414)	2,580 (1,843)	ND	5,750 (1,066)	ND

ND: not detectable, below the detection limit.

## Discussion

LIBS spectral features obtained in this study were in accordance with the expected atomic composition of the purchased protein powders and consistent with the results of another study^(23)^. They primarily contained atomic lines of alkali and alkaline earth metals (Na, Ka, Ca, and Mg), as well as lines from non-metallic elements, which are due to the protein content and other organic compounds. Atomic lines from toxic elements were not observed in any protein powder spectrum, confirming that their concentration was below the ppm level (the typical limit of detection in LIBS for most elements). The ICP-MS results confirmed that certain toxic elements (As, Be, Cd, Hg, Pb, and Sn) were in undetectable levels in the samples; however, in some cases, Al and Ba levels were increased. The vegan products contained higher Al and Mn levels, but lower Rb levels, than the nonvegan supplements.

Our results concur with those of the following reports: Elgammal et al. analysed 26 whey protein samples from Egypt and found Al, Cd, Pb, Sn, and Hg in some products (in 100.0%, 88.5%, 88.5%, 30.8%, and 0.0% fractions of the samples, respectively), which were at concentrations above the limit of quantification, yet below the safety levels^(24)^. Pinto et al. examined 49 whey products from the Portugal market and confirmed that these supplements were safe to use^(25)^. Bethencourt-Barbuzano et al. also supported the safety of normal protein supplement consumption; however, they cautioned that excessive consumption of supplements with Mo and Cr may pose a health risk^(26)^. Irshad et al. analysed 25 protein supplements from Pakistan and revealed that the composition of the products was within the safety threshold; however, consuming some supplements three times a day could exceed the tolerable weekly intake for Cd^(27)^. Philips et al. inspected 36 protein supplements from the Indian market and found Pb, Cd, and As in 75.0%, 27.8%, and 13.9% of the samples, respectively^(28)^. Supplements from India were found to have elevated Pb levels^(29)^. In the United Arab Emirates (UAE), dietary supplement manufacturers must ensure their products’ quality to gain access to the open market. Jairoun et al. examined 227 products for heavy metal content, and only 1.1% showed increased Cd, Pb, and/or As levels. Complex dietary supplements, which contain multiple ingredients, had a higher risk of heavy metal contamination than simpler products. Another research group from the UAE tested 200 health supplements in a cross-sectional study and could not detect As, Pb, Cd, and Hg in 93.0%, 94.5%, 100.0%, and 99.0% of the samples, respectively; only Cr exceeded the limit of detection in 76.5% of the samples^(30)^.

Due to the lack of mandatory quality assurance of food supplements, final products may contain contaminants at undetectable levels. However, a majority of consumers believe that these supplements are harmless^(31)^, and the general opinion is that they are safe and devoid of any adverse effects due to their organic ingredients.

The results of studies investigating other nutraceuticals are similar to those of this study. A study from Pakistan assessed the metal concentration in 20 coconut milk products, which is a popular dietary alternative to cow milk, and found varying amounts of nonessential metals in the products that may exceed the recommended dietary allowance limits^(32)^. Another study that investigated 50 dietary supplements revealed increased levels of Cr, Ni, and Cd, which were higher than the permissible level set by the World Health Organization; however, no adverse effects associated with the consumption of the products were reported^(33)^. Ahmed et al. analysed the metal components in 28 different beverages and 36 tea samples and found elevated levels of Ni and Cd, which posed a low risk of exposure^(34,35)^.

Protein and vitamin/mineral supplements are most commonly used for gaining strength and muscle mass or improving health^(36)^. Elite athletes tend to use more dietary supplements than non-professionals^(37)^. A study of adolescents showed similar patterns of use and reported that 44% of the participants believed that the use of protein supplements is not associated with any health risk^(38)^. The risk of heavy metal toxicity increases when supplements are consumed above the recommended level, especially by athletes, because the commonly recommended protein intake does not support the muscle growth they expect.

Similar to global trends, Hungary is also experiencing a growing market for protein powders^(39)^; however, factual information on the accurate quantities, market share of different products, and consumer habits is lacking. Literature highlights the importance of professionals raising consumer awareness about the potential risks associated with protein powders. These professional athletes have access to higher quality products and experts (e.g., doctors and dietitians) who can frequently guide and supervise their health; however, most of the common sport enthusiasts can only rely on the internet or their friends. Furthermore, consumers might purchase low-cost counterfeit products or products of unknown origin on the internet. Most of the tools that attempt to measure nutritional knowledge are outdated and lack validation. Nevertheless, it is alarming that in most studies, more than 50% of the adult athletes had inadequate nutritional knowledge^(40)^. The results are similar in the younger population, whose general nutritional knowledge is better than sports-related nutritional knowledge^(41)^.

### Conclusion

The results of this cross-sectional study demonstrated that heavy metal levels in protein powders available on the Hungarian market were low or undetectable. Nevertheless, consumers’ safety remains uncertain, given the caveat that the food supplement industry relies on voluntary compliance with food regulations. The findings of our study are not of local relevance, as the regulation of this product group is standardised across the EU; in addition, the products analysed were sourced from the EU, the USA, and Canada. Authorities should monitor foreign contamination (e.g. heavy metal, other substances) of protein powders, especially in the grey markets.

## Supporting information

Horváth et al. supplementary material Horváth et al. supplementary material

## Data Availability

The datasets used and/or analysed during this study are available from the corresponding author on reasonable request.

## Supplementary material

The supplementary material for this article can be found at https://doi.org/10.1017/jns.2025.10024
