# Supplementary material for: Analysis of heavy metal content in protein powders available on the Hungarian market: a reassuring snapshot, but not a reassuring quality guarantee
Source: J Nutr Sci. 2025 Jul 16;14:e49. doi: 10.1017/jns.2025.10024 (PMC12286494; doi:10.1017/jns.2025.10024)

**Analysis of heavy metal content in protein powders available on the Hungarian market: a reassuring snapshot, but not a reassuring quality guarantee**

István László Horváth1,2, Gyula Kajner 3, Gábor Galbács 3, Dezső Csupor 1,4,5 *

Affiliation

1 University Pharmacy Department of Pharmacy Administration, Semmelweis University, 1092, Budapest, Hőgyes Endre utca 7-9, Hungary

2 Center for Translational Medicine, Semmelweis University, 1085 Budapest, Üllői út 26, Budapest, Hungary

3 Department of Molecular and Analytical Chemistry, University of Szeged, 6720, Szeged, Dóm Square 7-8, Hungary

4 Institute of Clinical Pharmacy, Faculty of Pharmacy, University of Szeged, 6725 Szeged, Hungary

5 Institute for Translational Medicine, Medical School, University of Pécs, 7624 Pécs, Hungary

* Corresponding author

The detailed LIBS spectra of each investigated protein powders are found below.


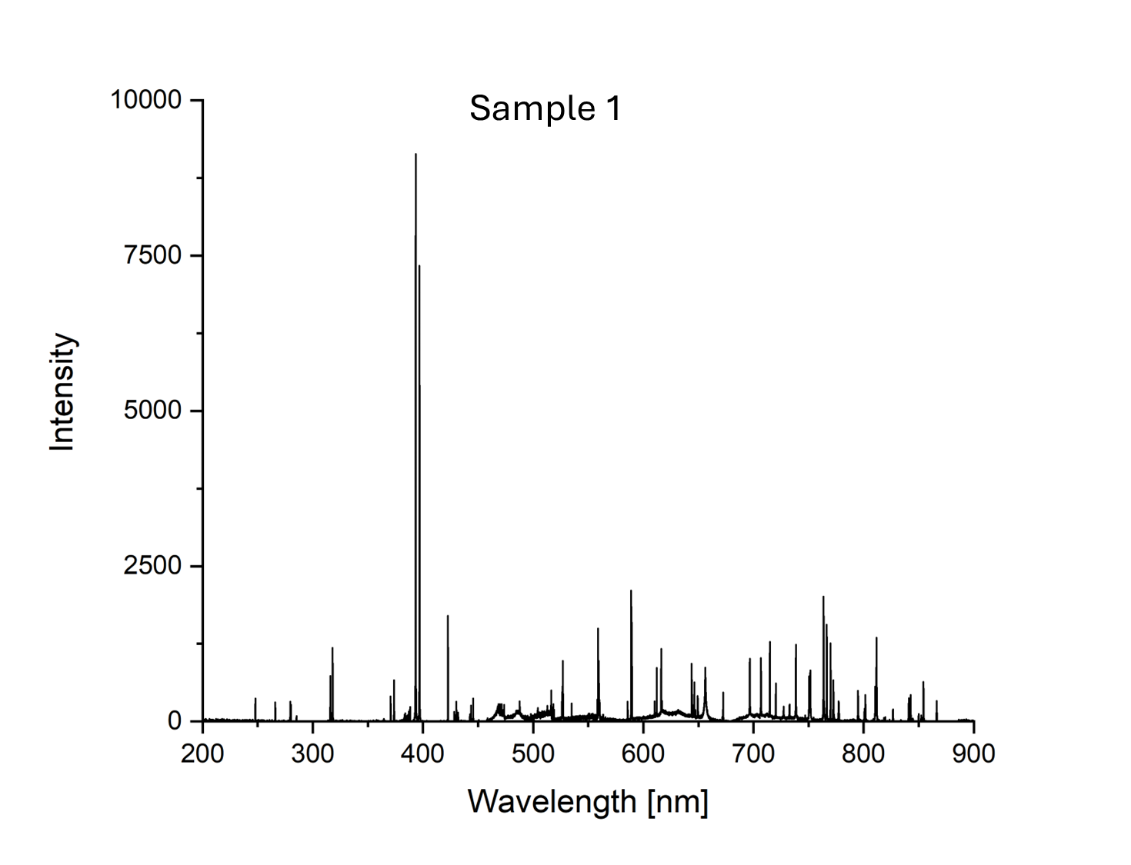


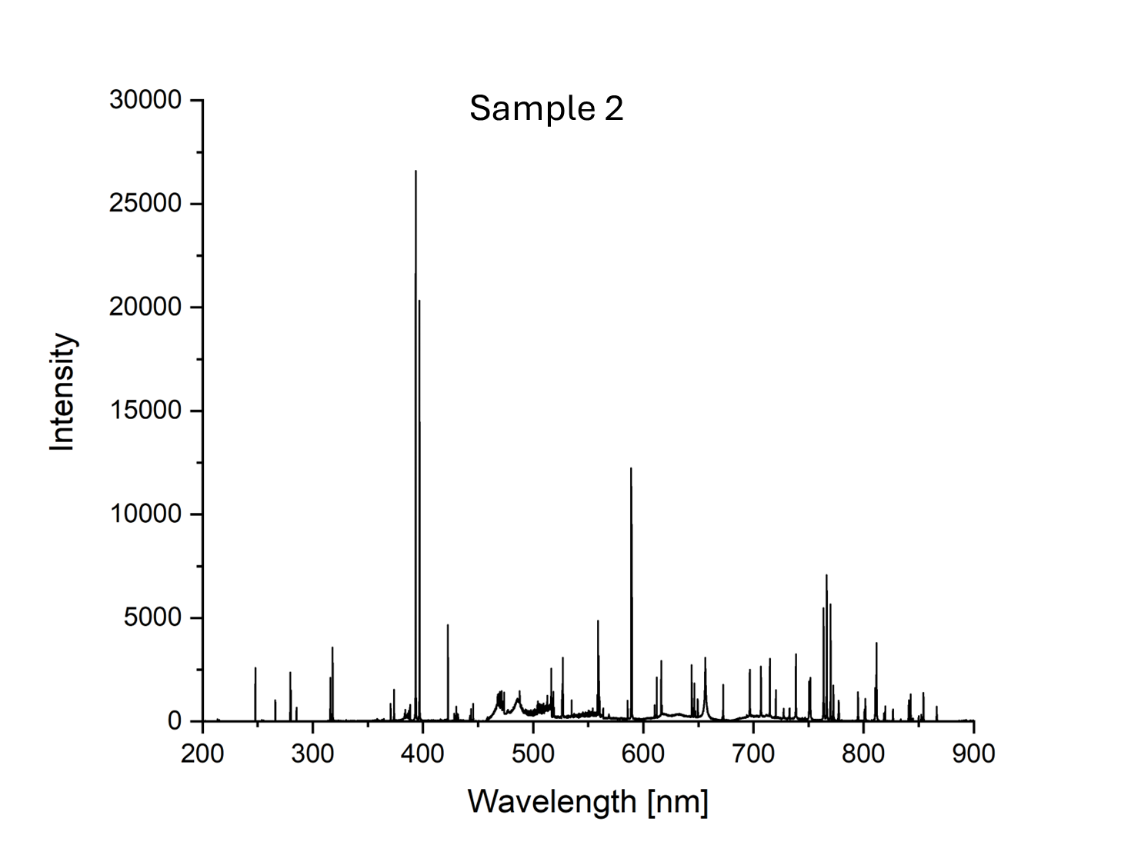


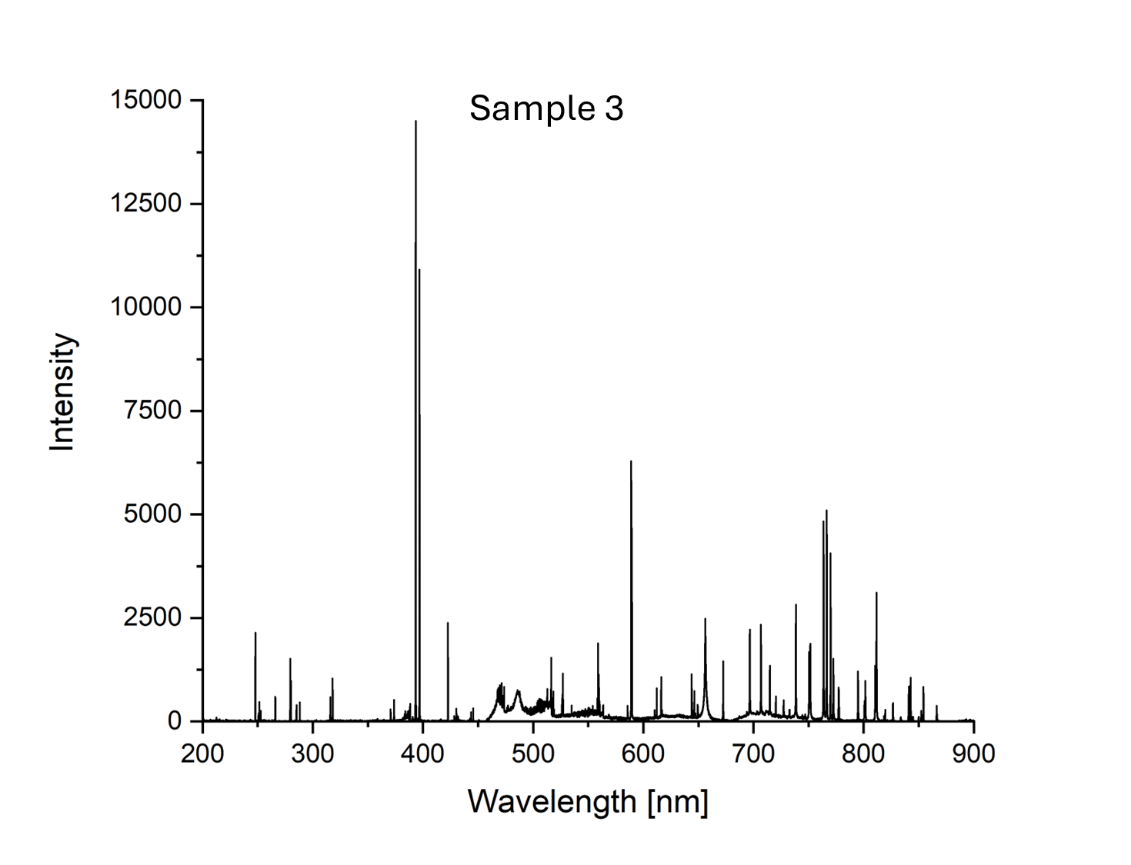


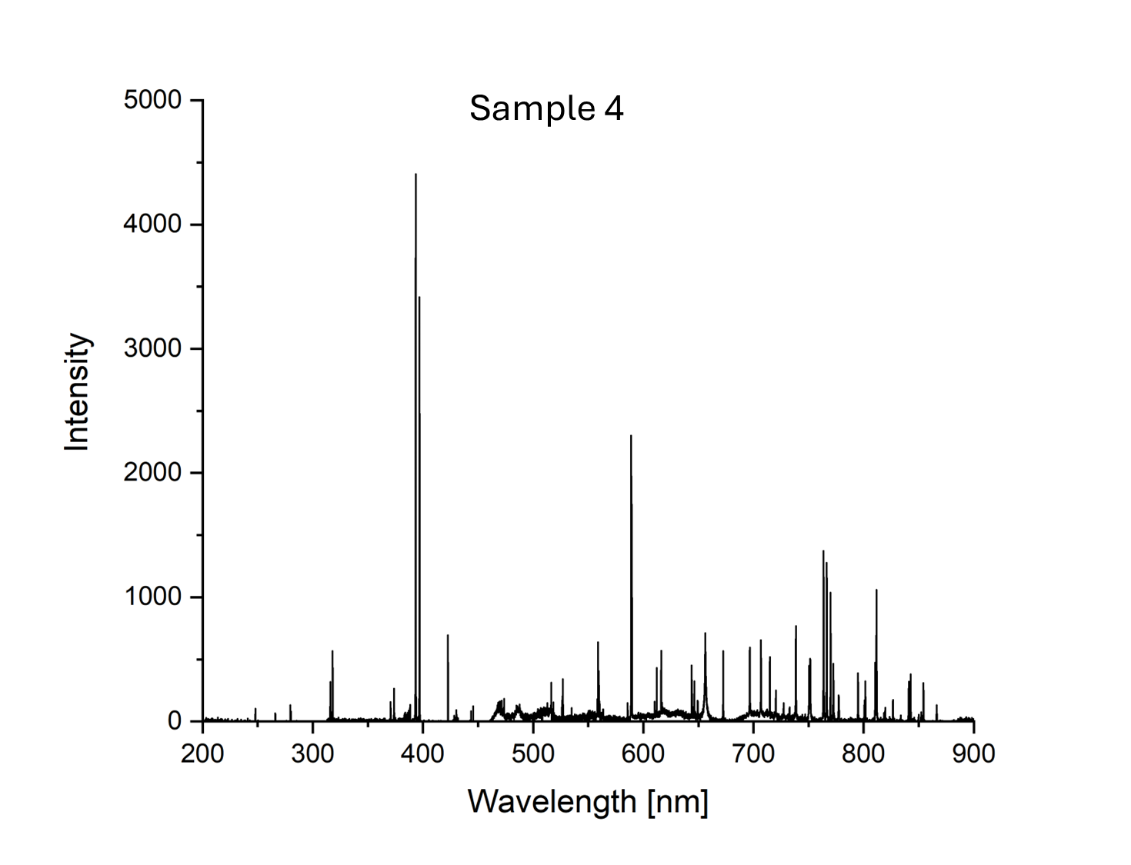


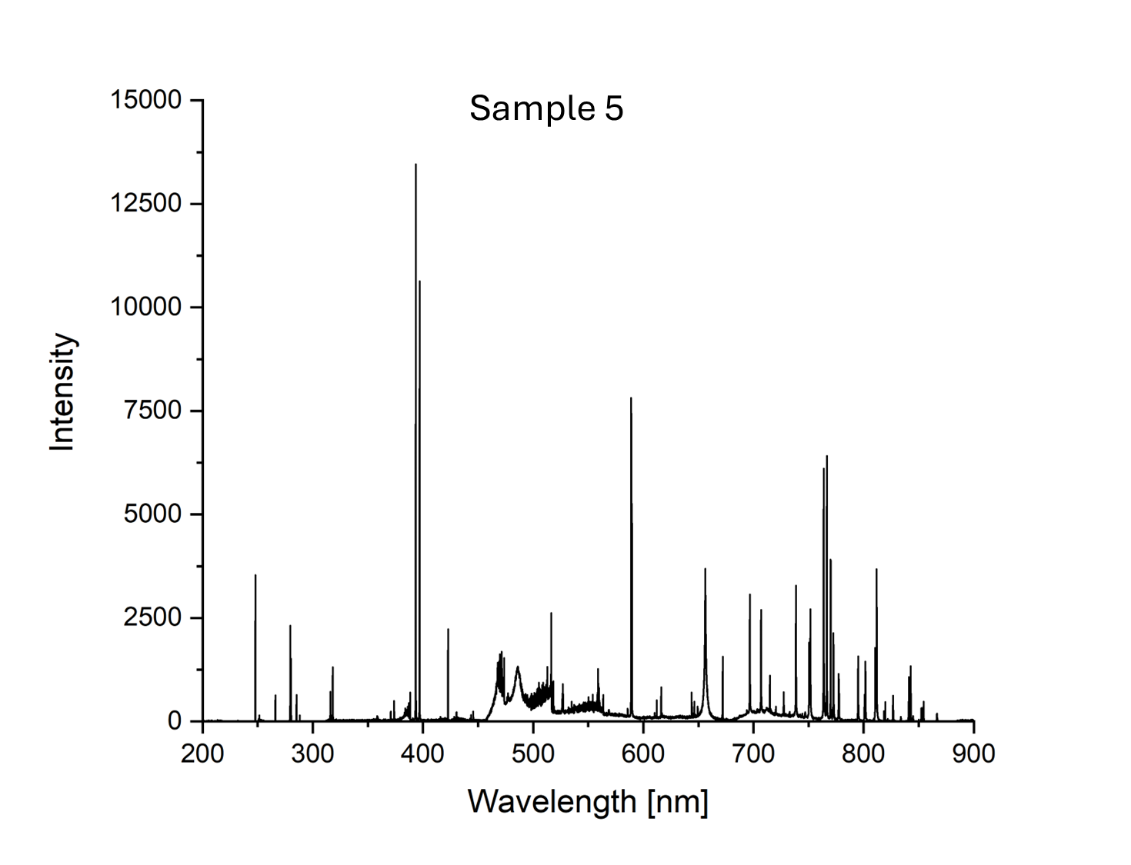


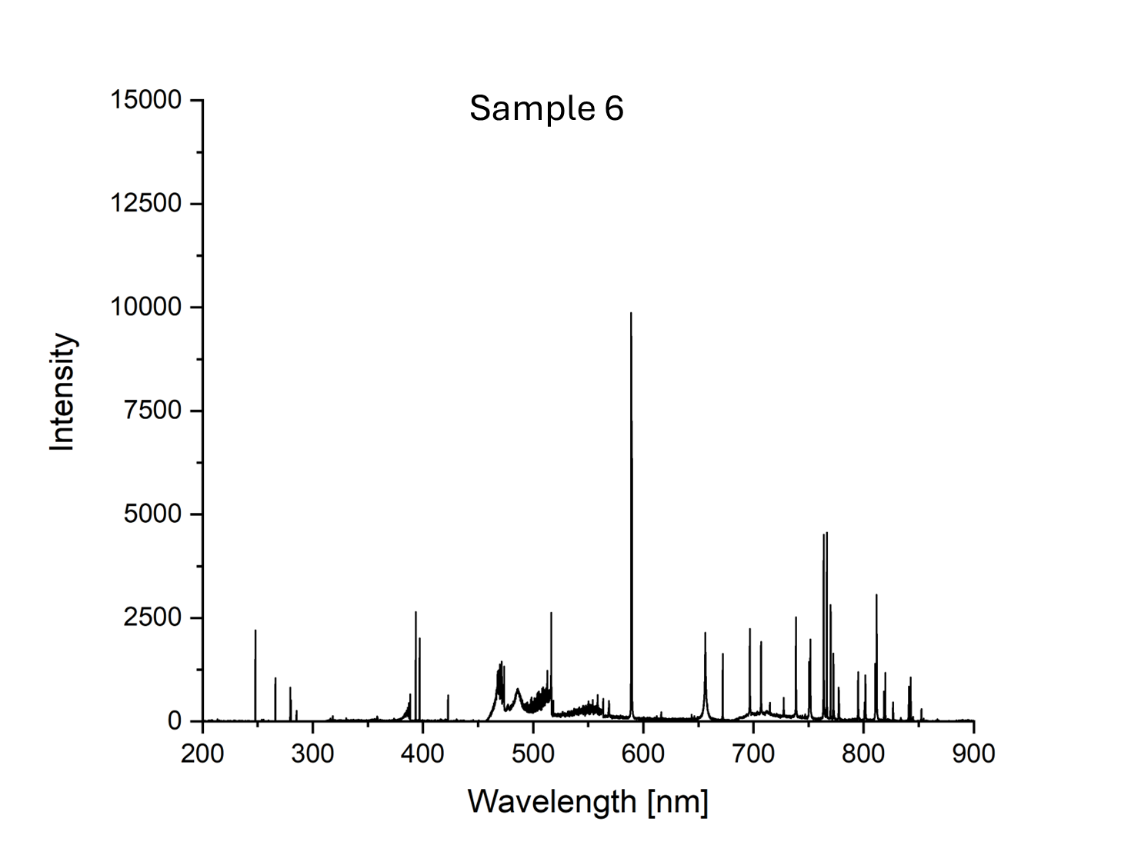


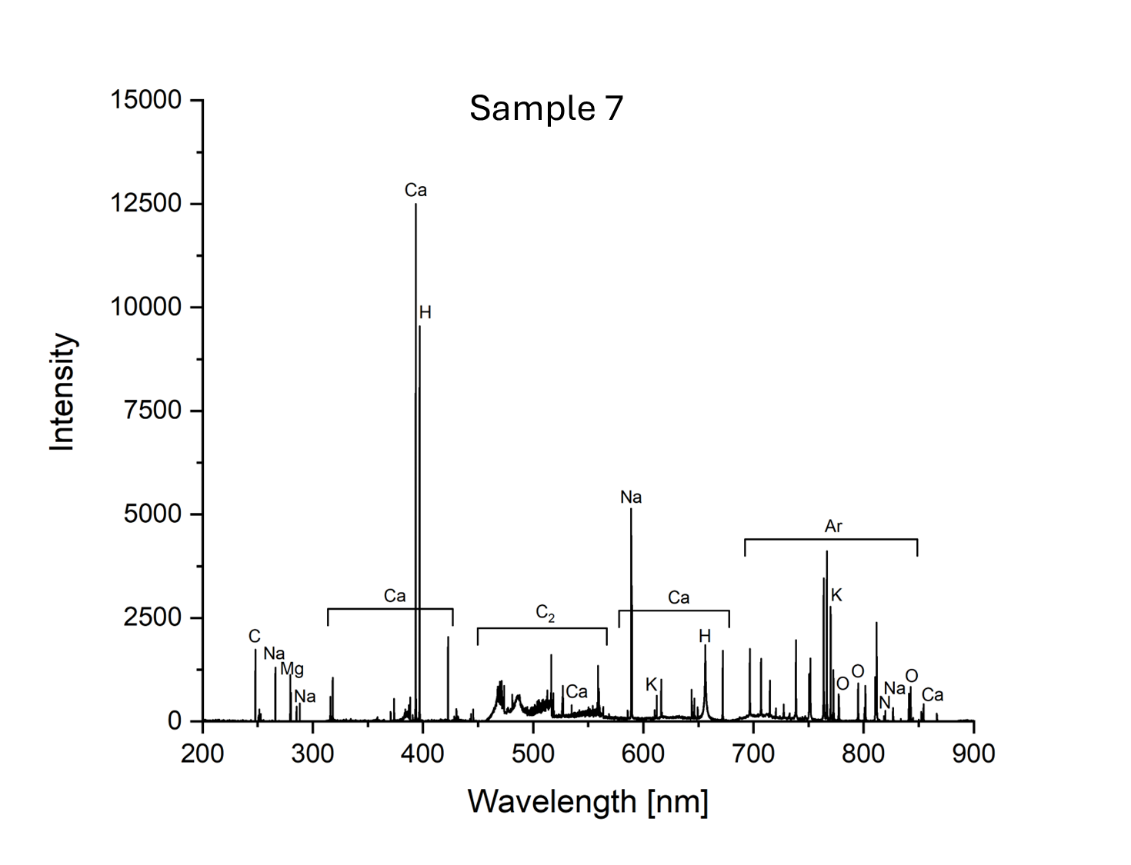


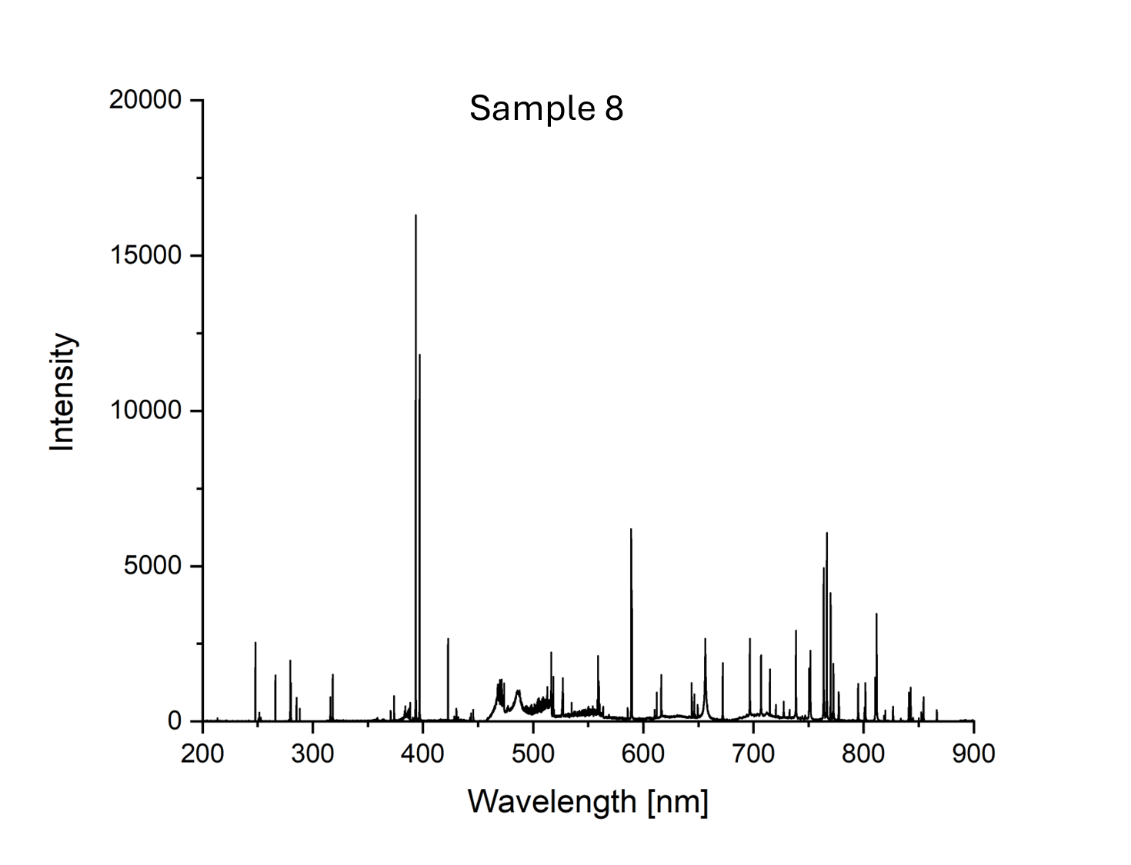


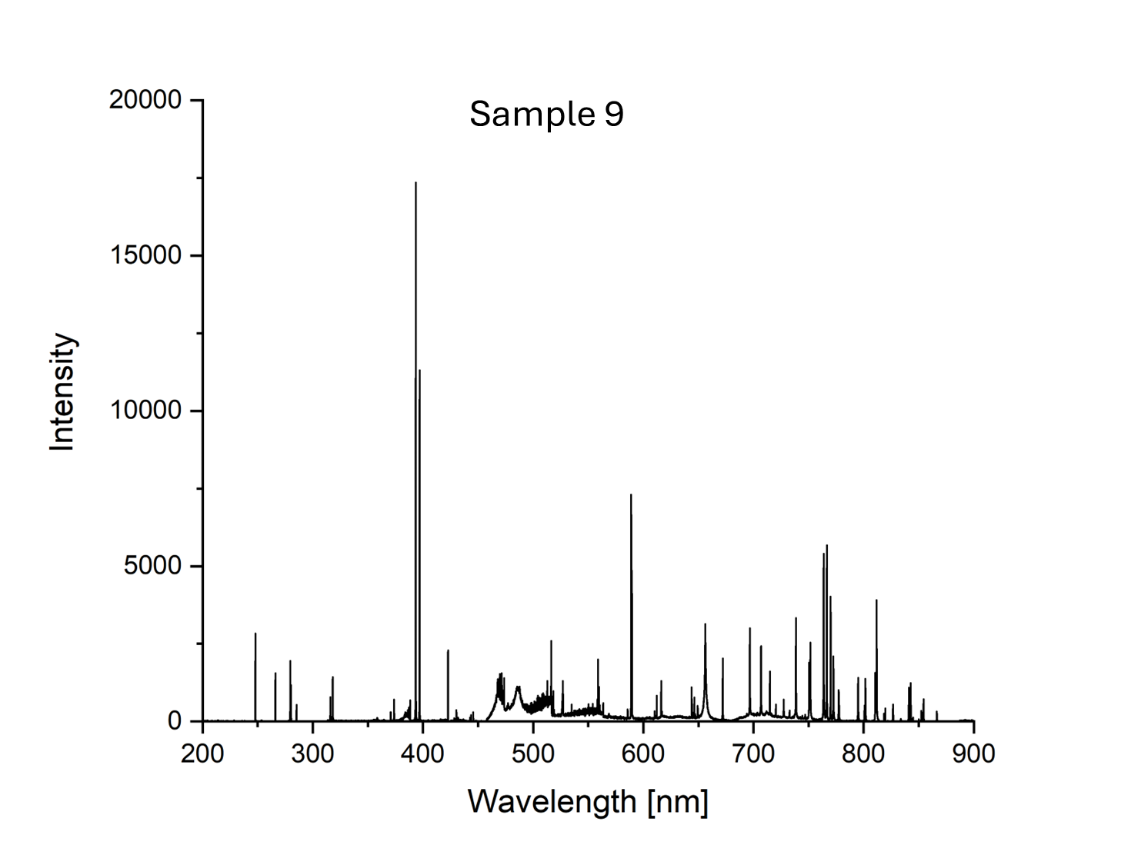


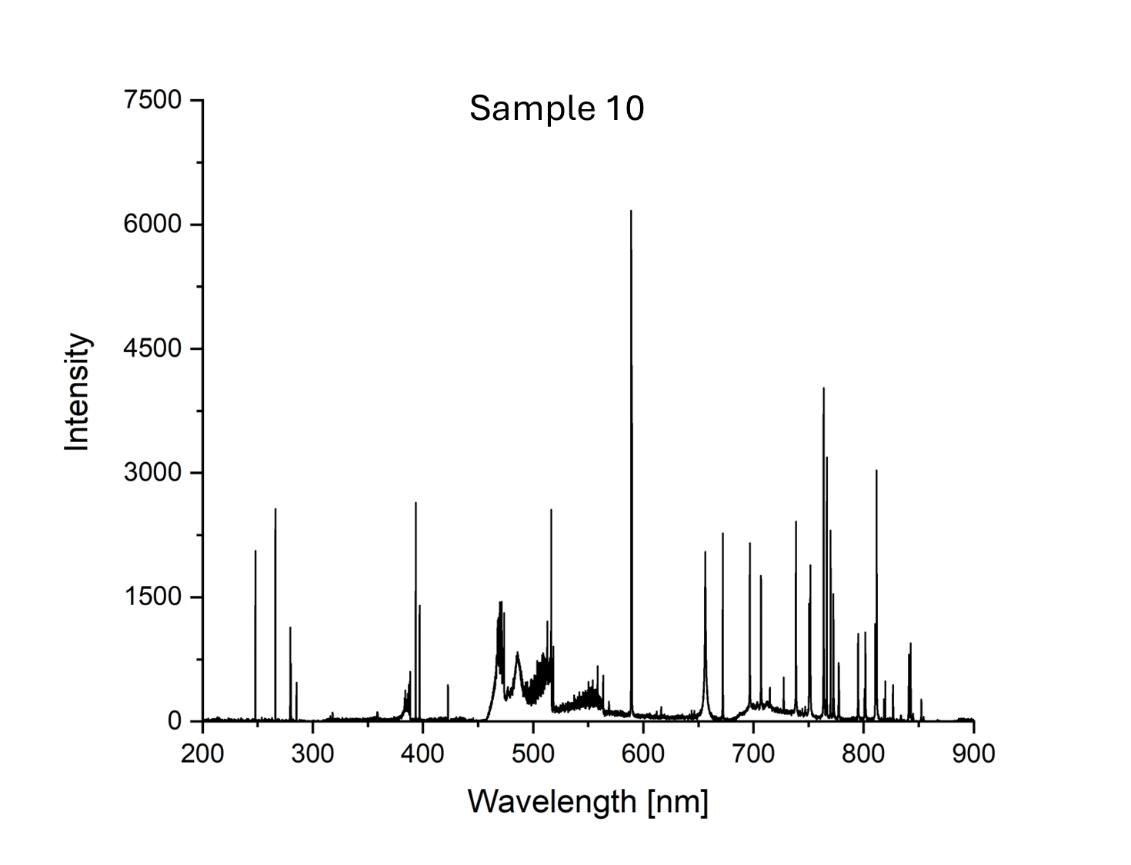


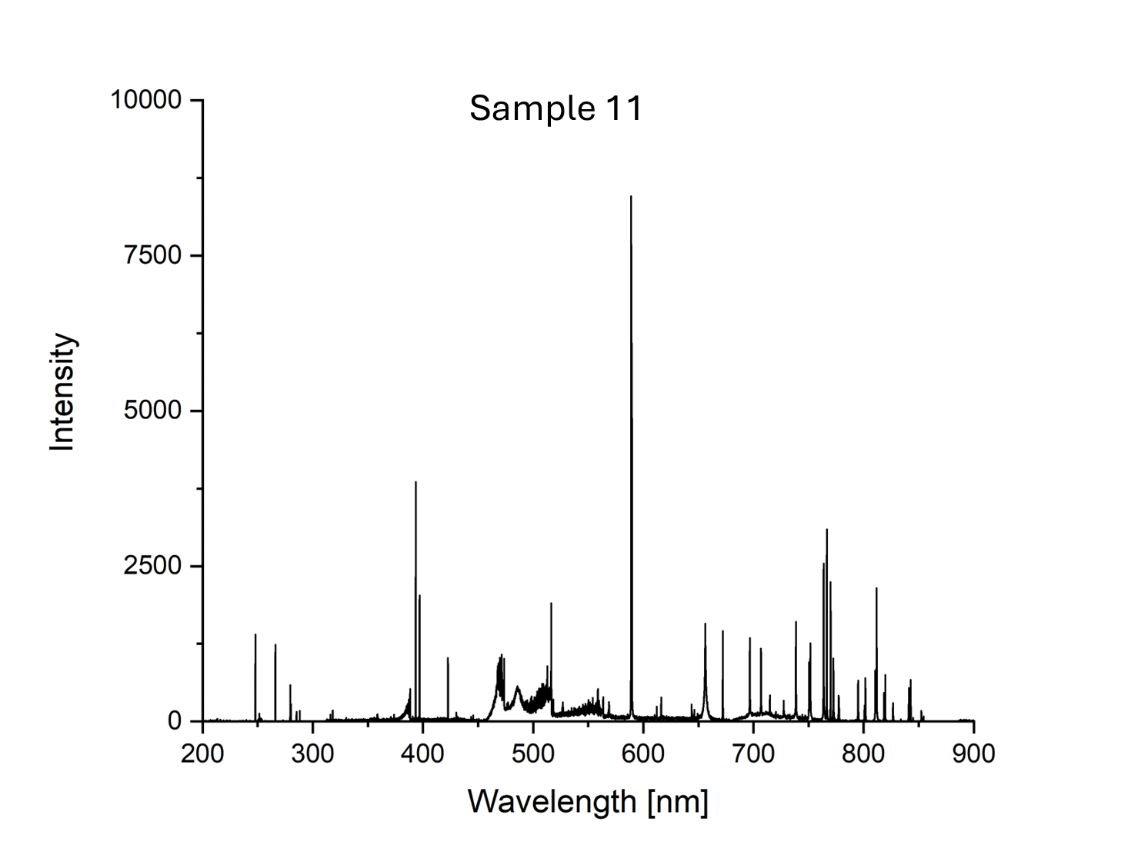


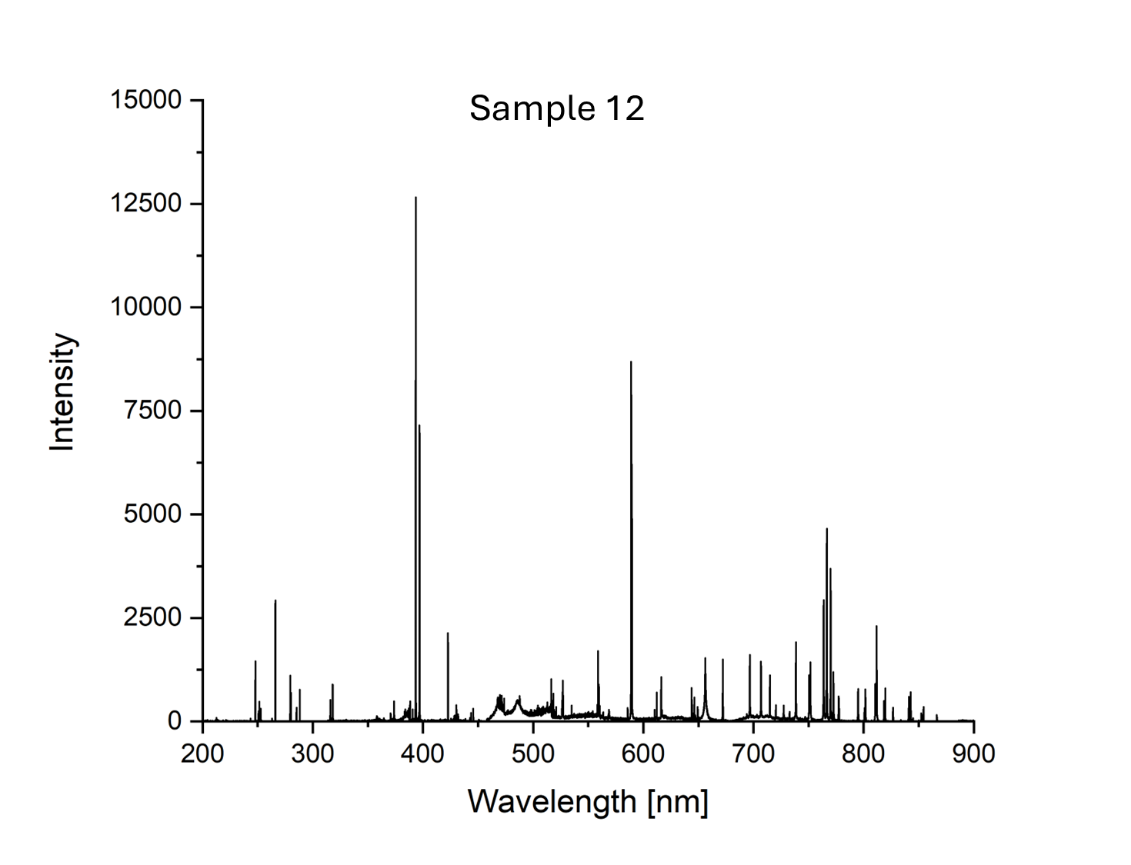


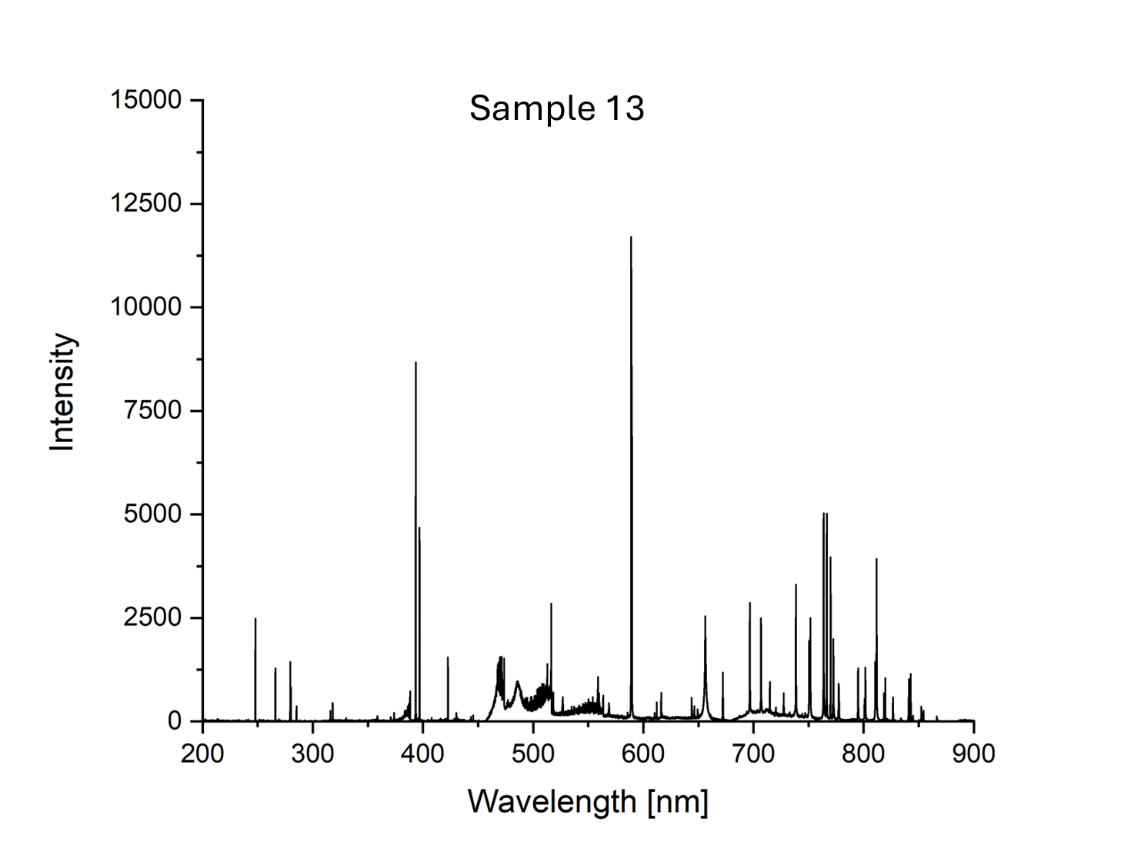


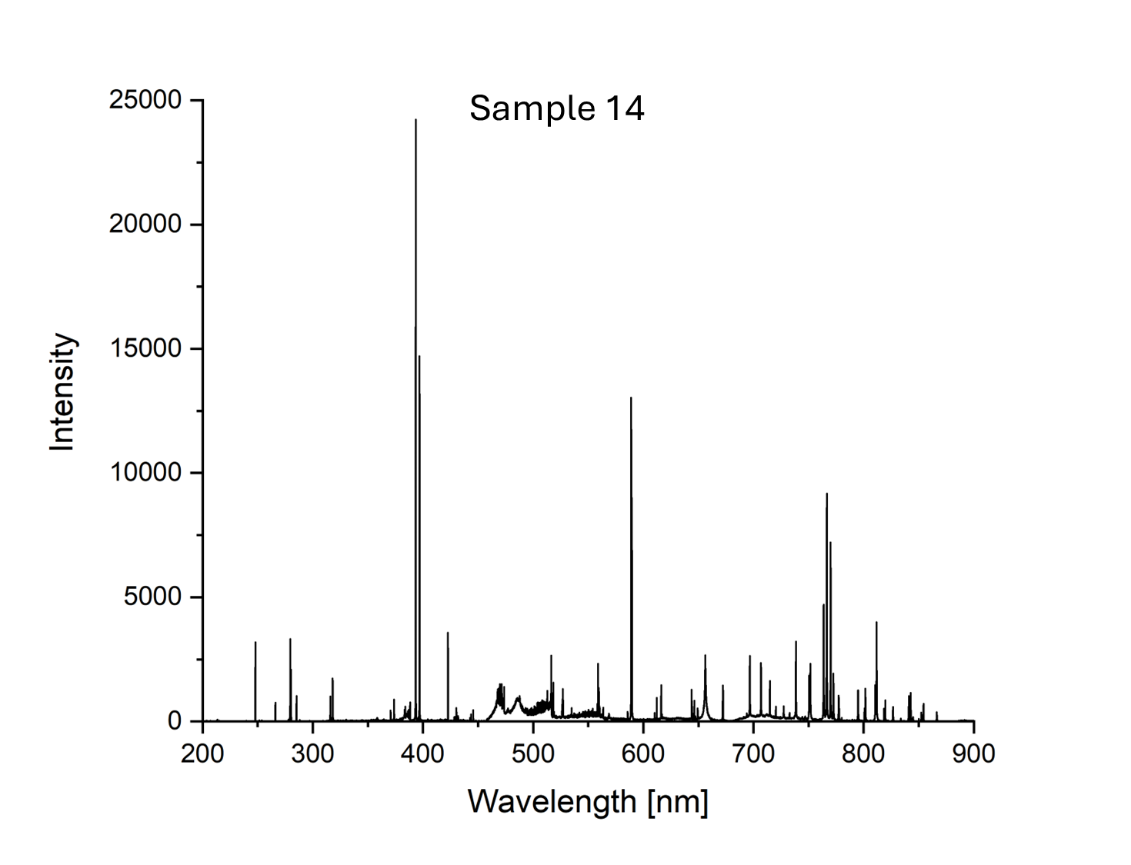


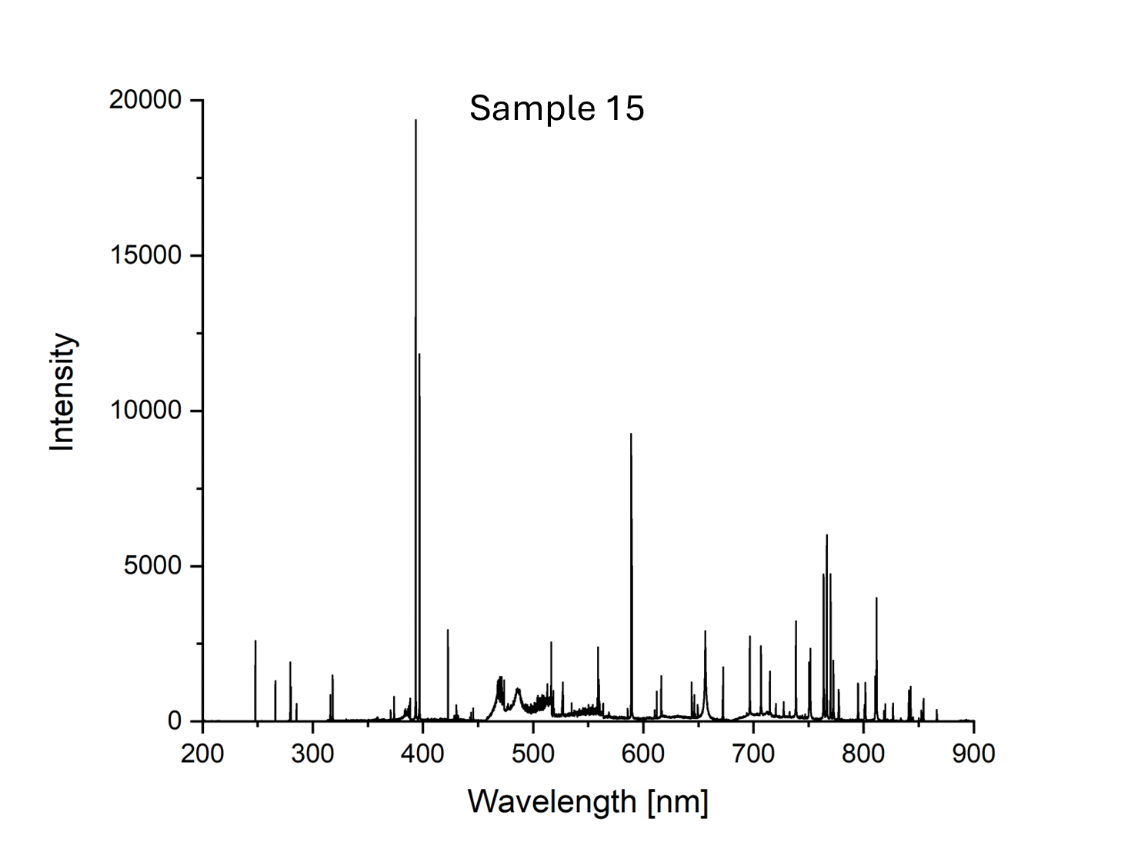


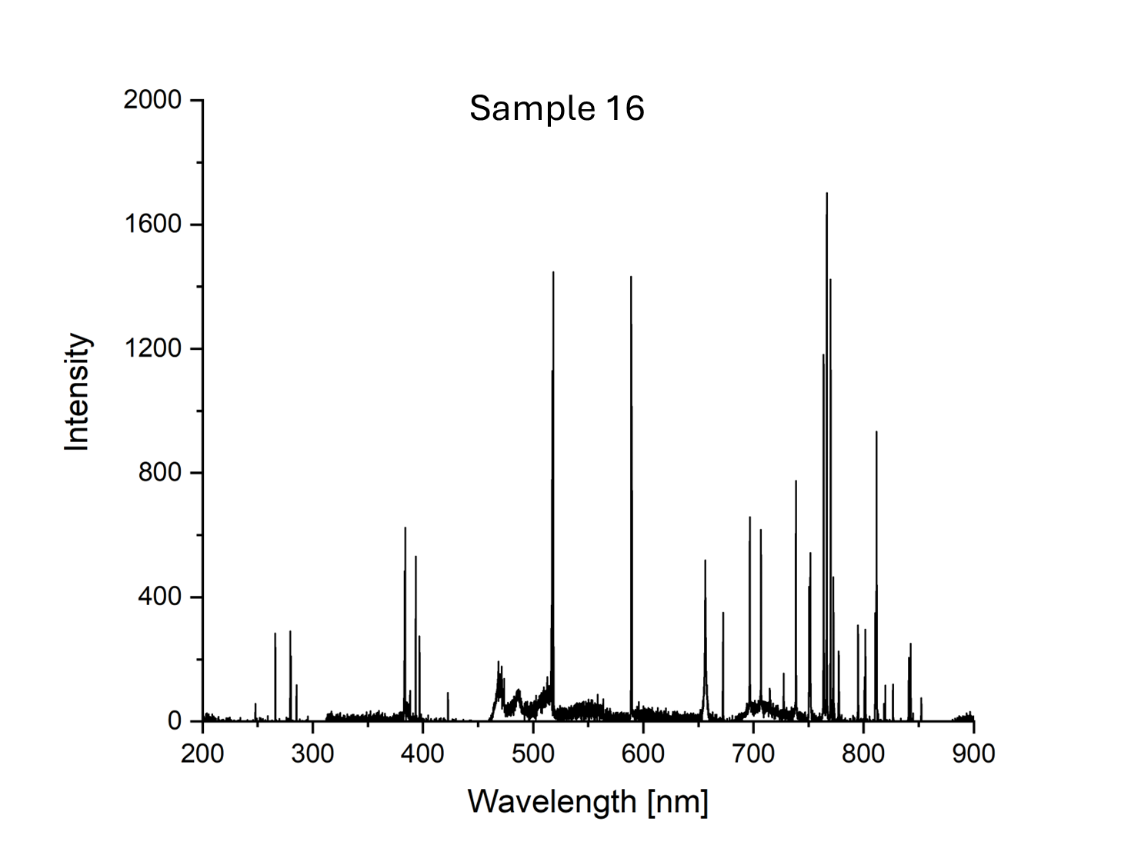


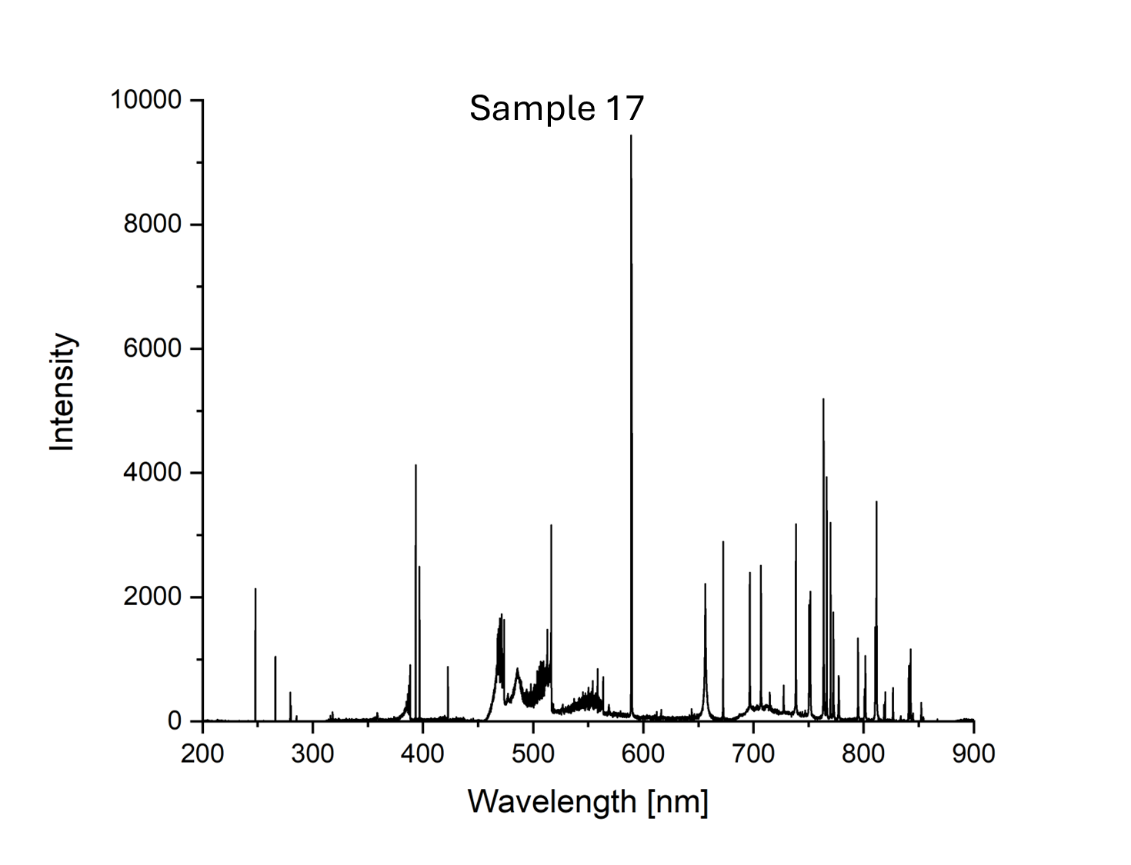


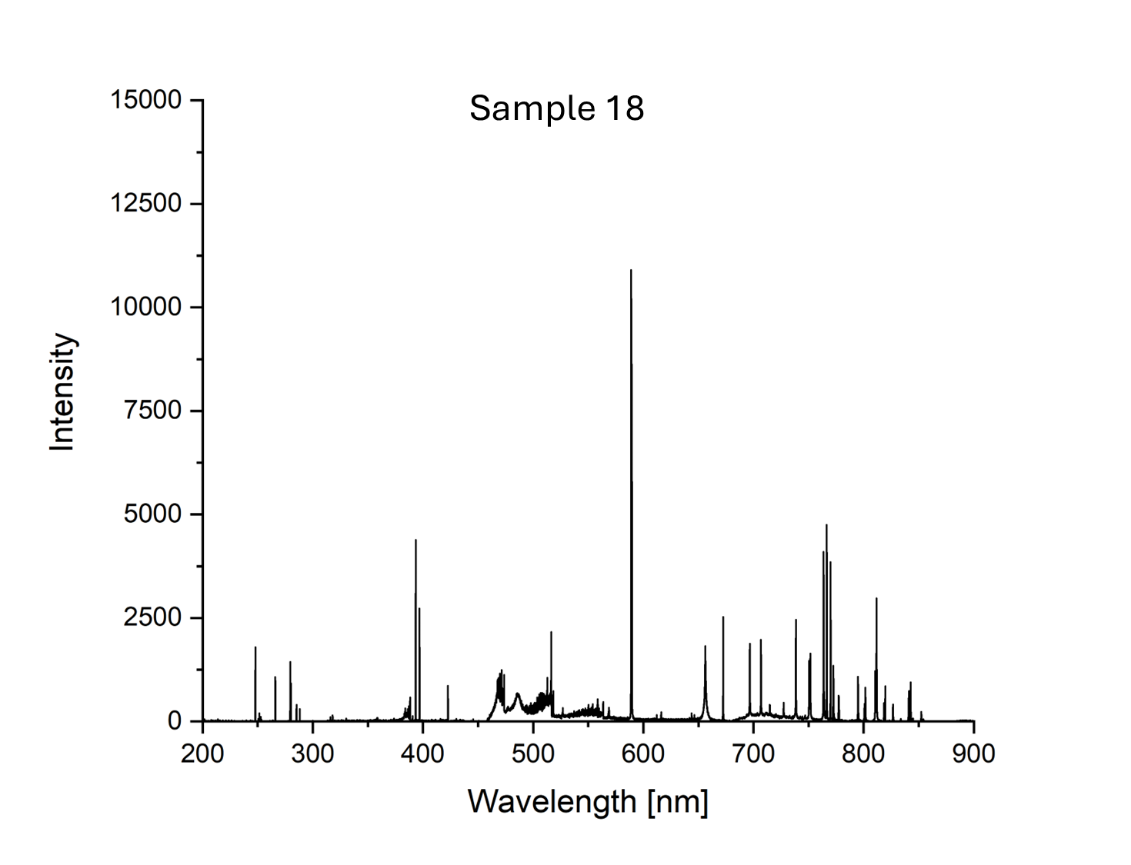


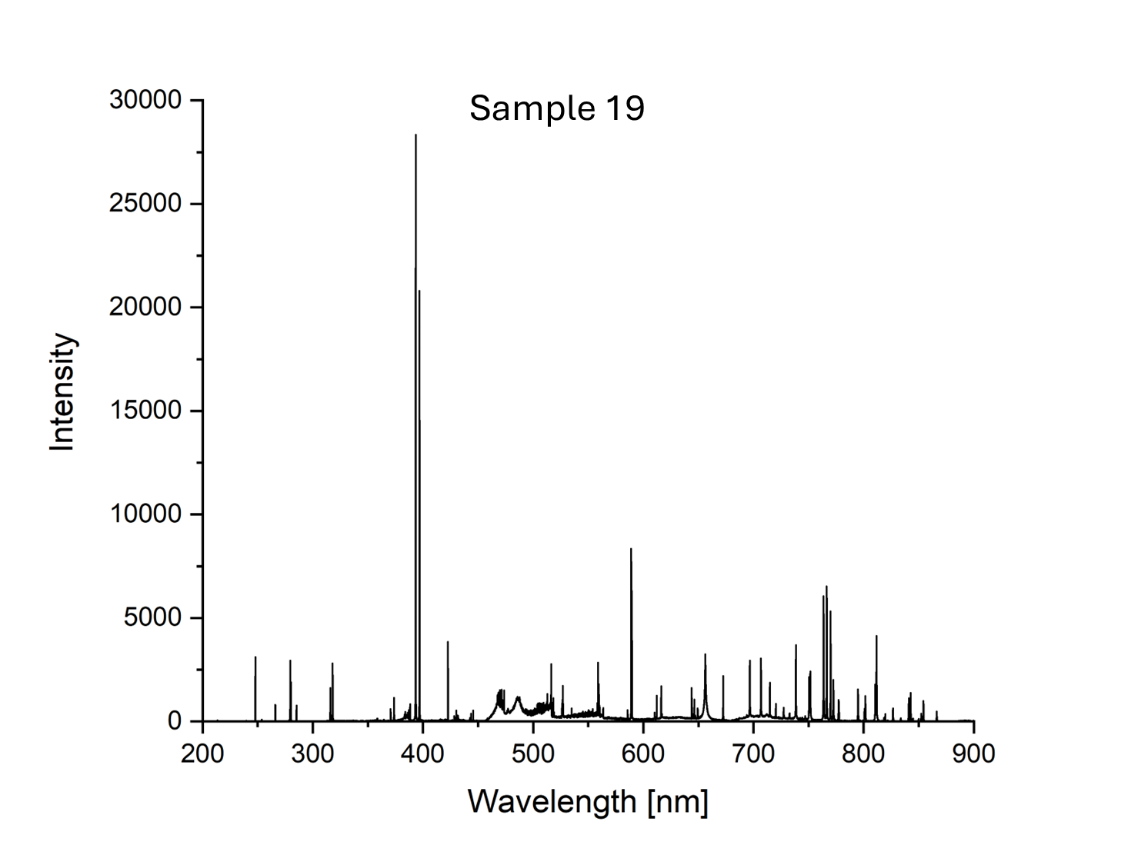


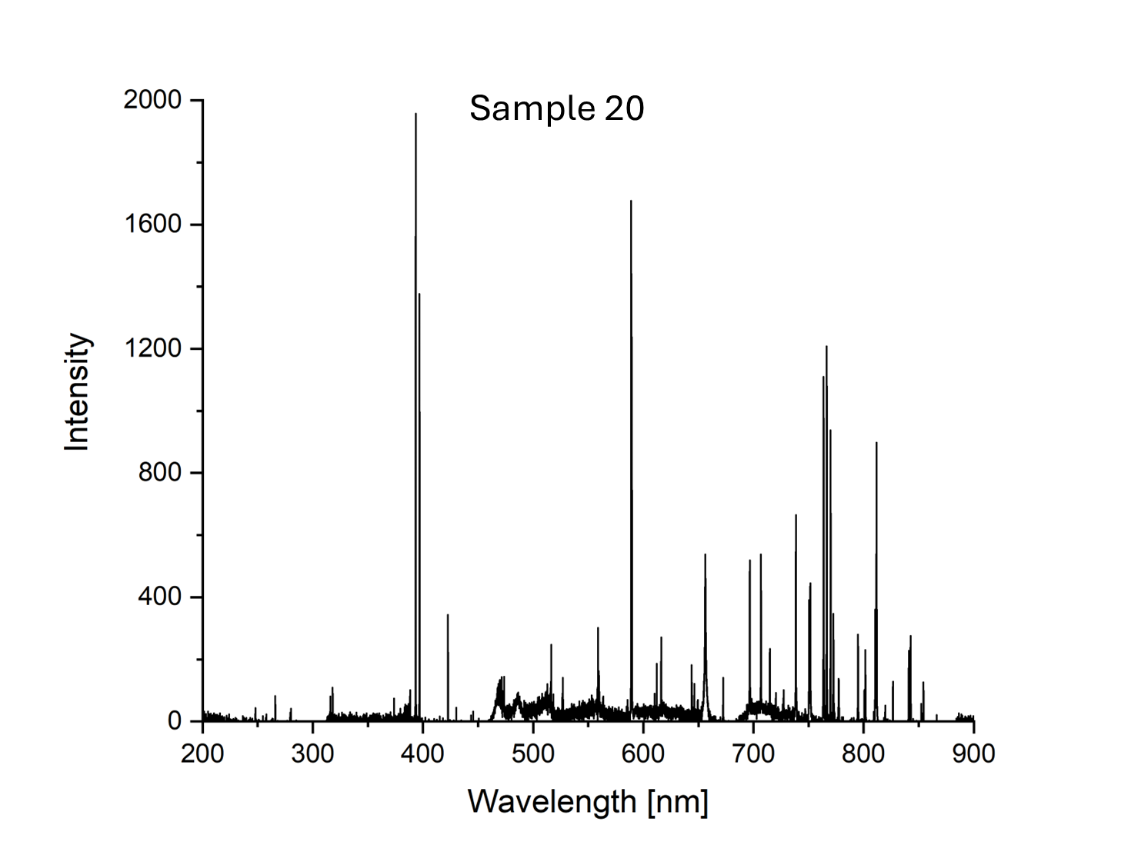


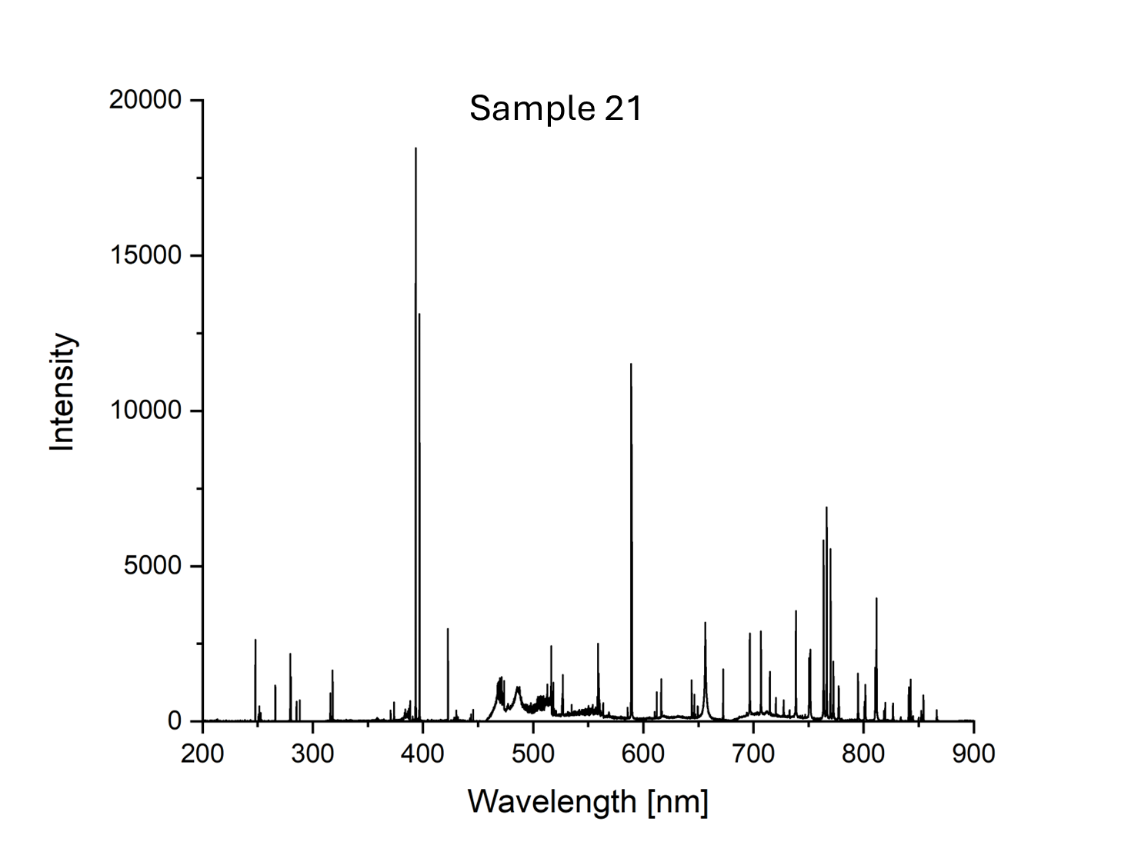


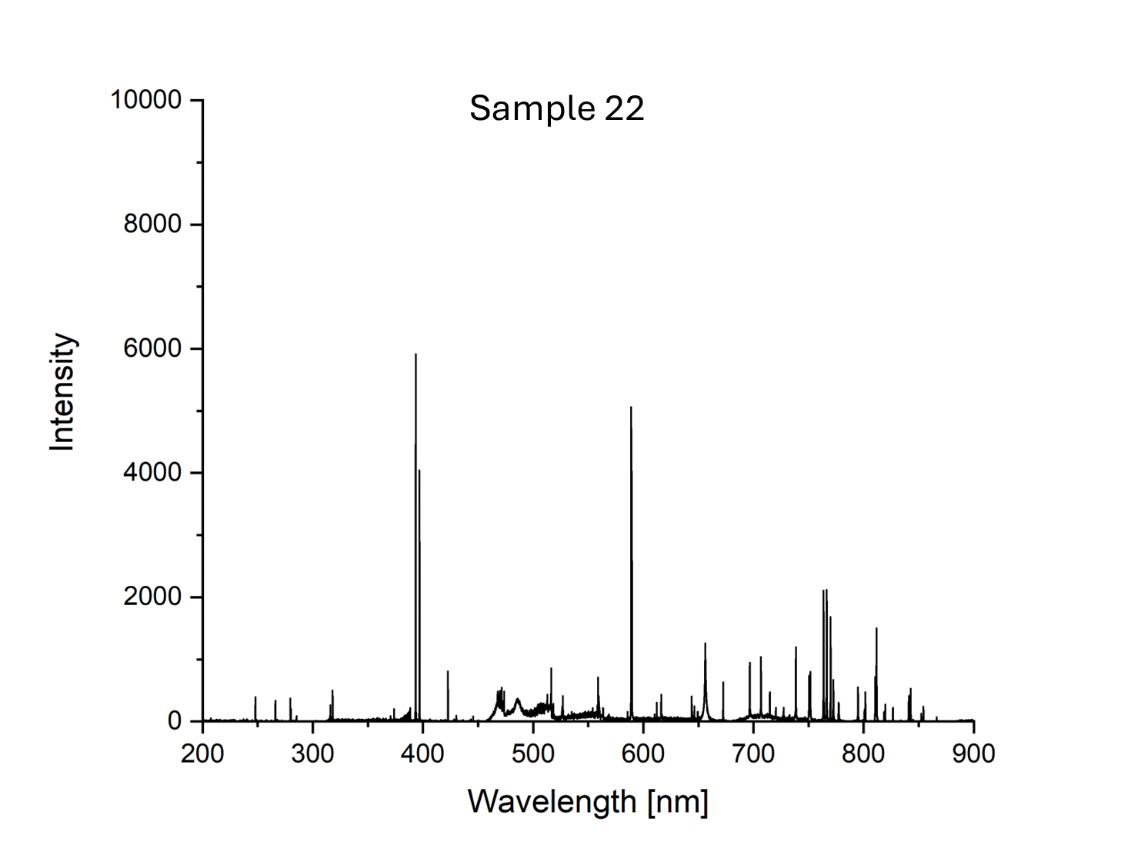

Supplement: Horváth et al. supplementary material [file S2048679025100244sup001.docx]
